# The prevalence of CRF55_01B among HIV-1 strain and its connection with traffic development in China

**DOI:** 10.1080/22221751.2021.1884004

**Published:** 2021-02-18

**Authors:** Mengze Gan, Shan Zheng, Jingjing Hao, Yuhua Ruan, Lingjie Liao, Yiming Shao, Yi Feng, Hui Xing

**Affiliations:** a State Key Laboratory of Infectious Disease Prevention and Control, National Center for AIDS/STD Control and Prevention (NCAIDS), Chinese Center for Disease Control and Prevention (China CDC), Collaborative Innovation Center for Diagnosis and Treatment of Infectious Diseases, Beijing 102206, China; b Guangxi Zhuang Autonomous Region Center for Disease Control and Prevention (Guangxi CDC), Nanning, China

**Keywords:** HIV-1, CRF55_01B strain, epidemic history, molecular network, phylogeographic

## Abstract

CRF55_01B is a relatively “young” HIV strain. At present, we do not know much about its transmission characteristics in China. So, to describe the transmission characteristics of CRF55_01B strain among provinces and HIV infected people, and to analyze the reasons for its rapid epidemic in China, a total of 1237 subjects infected with CRF55_01B from 31 provinces spanning a period of 12 years from 2007 to 2018 were enrolled in this study. By constructing a molecular network and Bayesian correlation analysis, we found that CRF55_01B increased exponentially from 2005 to 2009 after its origin in Shenzhen, and increased rapidly after 2010. CRF55_01B began to spread to other provinces in 2007. After 2010, the strain showed a trend of rapid spread and epidemic from Guangdong-Shenzhen to other provinces in China. Guangdong, Shenzhen, Hunan, Beijing, Guangxi, Hubei, Jiangxi, Guizhou, Hebei, Anhui, Shanghai, Shandong, Henan, and Yunnan were the key provinces of CRF55_01B transmission. CRF55_01B, although originating from men who sex with men (MSM), was transmitted among heterosexuals in 2010. Males in heterosexuals played a crucial role in the transmission and diffusion of this strain. We also revealed that CRF55_01B might spread rapidly along with the rapid development of the Beijing-Guangzhou and Beijing-Kowloon railways. This study suggests that if we detect the spread of MSMs in time through molecular monitoring in the early stage of the epidemic, it can help us control the epidemic early and prevent its spread, which is of great significance to China's national prevention and control of HIV-1.

## Introduction

In the 35 years of the epidemic in China, HIV-1 has formed many Circulating Recombinant Forms (CRFs) strains [1–3]. CRF55_01B of HIV-1 strain has attracted much attention in recent years as it is the first CRF01_AE and B subtype recombinant strain in China. It is also the first CRF identified in China in this century since CRF07_BC and CRF08_BC were discovered last century. CRF55_01B was first reported in Changsha, Hunan province, and Dongguan, Guangdong Province in 2013 [4] and has been detected in many cities in China since it was identified [5].

CRF55_01B was first discovered in men who sex with men (MSM). Previous studies have shown that CRF55_01B originated around 2000–2003 and mainly spread in MSMs, and then rapidly spread throughout the country [5–7]. CRF55_01B stood out among CRF01_AE and B subtype recombinant strains (CRF59_01B [8], CRF67_01B, CRF68_01B [9]) discovered in the same year, as well as many new CRFs (CRF79_0107 [10], CRF80_0107 [11], etc.) found in MSMs and became the fifth most prevalent strain of HIV-1 in China [12]. Therefore, this study will focus on the analysis of how CRF55_01B rapidly spread to the whole country in a short time and the characteristics of transmission among provinces and infected people.

## Materials and methods

### Study population

All available CRF55_01B *pol* gene region sequences (HXB2 : 2253-3312nt, 1060 bp) were collected from the HIV sequence database of the Los Alamos National Laboratories (LANL) and the National Center for AIDS/STD Control and Prevention of China (NCAIDS). RaxmlGUI v2.0.0 [13] was used to construct the RaxML phylogenetic tree for subtype identification and eliminate duplicates and sequences without province and sampling year information. The demographic data missing was mainly in the sequences of Guangdong. Since there were too many unknown demographic data sequences in the data set, two data sets were established. Data set A contained all sequences for the molecular network analysis. Data set B was another database built according to the priority order of region, province, sampling year, risk, sex, and age for Bayesian analysis (**Table S1**).

### Phylogenetic analysis and HIV-1 molecular network construction

A molecular network was constructed using HIV-TRACE (Transmission Cluster Engine) [14]. We aligned HIV *pol* sequences to an HXB2 reference sequence and calculated pairwise genetic distances under the Tamura-Nei 93 model [15]. The ambiguous nucleotides of all sequences were less than 5%. Each individual in the molecular network was represented by a node, and we linked nodes to each other if their pairwise genetic distance was up to 0.5% substitutions per site based on the recommended genetic distance threshold by the Centers for Disease Control and Prevention (CDC) in the United States (US) [16].

### Time-scaled phylogenetic tree reconstruction using BEAST

To reconstruct the temporal and spatial dynamics of CRF55_01B strain in various provinces, we performed a Bayesian discrete phylogeographic approach to estimate the rate of evolution and the time to the most recent common ancestor (tMRCA) using Markov chain Monte Carlo (MCMC) runs of 200 million generations with BEAST v1.8.4 [17] under a Bayesian Skygrid demographic model [18]. The final data set was analyzed using a general time-reversible (GTR) nucleotide substitution model [19] specifying a gamma distribution as a prior on each relative substitution rate and a relaxed uncorrelated lognormal (UCLN) molecular clock model to infer the timescale of HIV-1 evolution with a gamma distribution prior on the mean clock rate (shape = 0.001, scale = 1000) [20,21]. The Bayesian MCMC output was analyzed using Tracer v1.6 [22]. The Effective Sample Size (ESS) values for estimates were more than 200. Using LogCombiner (in BEAST package), we subsampled the posterior distribution of phylogenetic trees to generate an empirical distribution of 2000 trees representative of the posterior sample. The first 10%–30% of the states from each run were discarded as burn-in. Trees were summarized as maximum clade credibility (MCC) trees using TreeAnnotator (in BEAST package) and then visualized in FigTree v1.4.4 (http://tree.bio.ed.ac.uk/software/figtree). SpreaD3 v0.9.7.1 [23] was used to draw the CRF55_01B propagation roadmap.

### Discrete phylogeographic analyses and spatial structure

To provide an adequate description of the process of viral dissemination, we use a Bayesian stochastic search variable selection (BSSVS) [24] procedure. We expected to analyze the relationship between transmission risk groups (Risk), risk and sex (Risk-Sex), and risk and age (Risk-Age). We also used a robust counting (Markov jumps) [25] approach to count the expected number of virus lineage movements. Statistical support was measured using Bayes factors (BF) [24] and summarized using SpreaD3 [23].

### Discrete trait and a Bayesian Tip-association Significance Testing (BaTS)

A Bayesian Tip-association Significance Testing (BaTS) provided a method by which the degree to which traits seen in a phylogeny are associated with ancestry are correlated [26]. To evaluate phylogenetic correlations between provinces, we estimated the phylogenetically based Association Index (AI) statistic, Parsimony Score (PS) statistic, and Monophyletic Clade (MC) statistics for each discrete-trait using BaTS v0.9 beta.

The AI and PS statistics tested the association between provinces and tree topology, considering the level of uncertainty in the phylogenetic reconstruction. The MC index tested which traits (provinces) were associated with phylogeny. The observed mean and its associated 95% confidence intervals (Upper and Lower CI) were obtained by analyzing trees sampled during the Bayesian phylogenetic reconstruction. The null mean and associated confidence intervals were obtained after randomly distributing the phylogeny traits (100 replicas). The significance level was the *P*-value for the statistical hypothesis test for equality between the index observed and expected under no association [27].

### Statistical analysis

Cochran–Armitage was used to analyze the variation trend of transmission risk groups over time. According to whether they belong to a molecular network, a comparison of demographic characteristics was based on chi-square tests. Fisher test was used when the number of cells was less than 5. Wilcoxon rank-sum test was used for Beijing-Guangzhou and Beijing-Kowloon railways analysis. *P* < 0.05 was statistically significant. All statistical tests were performed using R v4.0.2.

## Results

### Demographic characteristics of CRF55_01B

A total of 1237 (734 from LANL database and 503 from NCAIDS database) sequences sampling time spaned from 2007 to 2018 were obtained for the subsequent analysis. The data set covers 31 provinces mainly distributed in Guangdong, especially in Shenzhen (Shenzhen, belongs to Guangdong) followed by the neighboring Guangxi in south China, Beijing in North China, and Hunan in central China. To increase the analysis’s resolution, as the origin of CRF55_01B, and due to a large number of sequences, Shenzhen was listed separately in all the following analyses. MSMs are the main transmission route of the research subjects in (Table 1). Although CRF55_01B was first found in MSMs, it gradually spreads to heterosexuals. By using the chi-square trend test, it was found that heterosexuals showed an increasing trend in three periods: 2007–2012, 2013–2015, and 2016–2018 (Table 2).

**Table 1.** Data set A – Demographic characteristics of CRF55_01B.

	Overall	Risk				Sampling year	Provinces (n)
		MSM	HET	IDU	Unknown		
North	109	59	12	0	38	2008–2018	BJ (75), TJ (3), HE (21), SX (5), NM (5)
Northeast	10	8	1	0	1	2014–2018	LN (4), JL (3), HLJ (3)
East	135	102	27	1	5	2011–2018	SH (32), JS (24), ZJ (11), AH (19), FJ (6), JX (16), SD (27)
Centre	103	58	40	0	5	2009–2018	HA (13), HB (22), HN (68)
South	813	262	102	8	441	2007–2018	GD (454), SZ (283), GX (71), HI (5)
Southwest	42	17	22	1	2	2009–2018	CQ (10), SC (7), GZ (12), YN (11), XZ (2)
Northwest	25	14	10	0	1	2012–2018	SN (16), GS (3), QH (1), NX (3), XJ (2)
Overall	1237	520	214	10	493	2007–2018	31 provinces

Abbreviations: **Risk:** HET, heterosexual. MSM, men who have sex with men. IDU, injecting drug users. **Provinces:** Anhui (AH), Beijing (BJ), Chongqing (CQ), Fujian (FJ), Guangdong (GD), Gansu (GS), Guangxi Zhuang Autonomous Region (GX), Guizhou (GZ), Henan (HA), Hubei (HB), Hebei (HE), Hainan (HI), Heilongjiang (HLJ), Hunan (HN), Jilin (JL), Jiangsu (JS), Jiangxi (JX), Liaoning (LN), Inner Mongolia Autonomous Region (NM), Ningxia Hui Autonomous Region (NX), Qinghai (QH), Sichuan (SC), Shandong (SD), Shanghai (SH), Shaanxi (SN), Shanxi (SX), Shenzhen (SZ), Tianjin (TJ), Xinjiang Uygur Autonomous Region (XJ), Tibet Autonomous Region (XZ), Yunnan (YN), Zhejiang (ZJ).

**Table 2.** The change of transmission risk groups with time in CRF55_01B

	Overall (%)	2007–2012 (%)	2013–2015 (%)	2016–2018 (%)	Z-value	*P*-value
Risk^＊^	1237 (100.0)	427 (34.5)	493 (39.9)	317 (25.6)		
MSM	520 (42.0)	212 (49.6)	144 (29.2)	164 (51.7)	0.13	0.90
HET	214 (17.3)	32 (7.5)	70 (14.2)	112 (35.3)	9.66	<0.0001
Others	503 (39.9)	183 (42.9)	279 (56.6)	41 (12.9)	7.31	<0.0001

＊: Others include IDU and Unknown. HET, heterosexual; MSM, men who have sex with men; IDU, injecting drug users; Unknown, we do not known their risks.

### Molecular network analysis of CRF55_01B

Under the threshold of 0.5% genetic distance, 60.5% (748/1237) sequences (nodes) from a total of 98 clusters were enrolled in the molecular network. The largest cluster consists of 46.3% (346/748) nodes, including 20 provinces. The molecular network diagram of CRF55_01B is shown in **Figure S1**. Whether infected people belong to the molecular network is related to risk, age, sampling year, region, and province (**Table S2)**.

65.2% (488/748) of the nodes in the molecular network are from Guangdong-Shenzhen. 70.9% (3106/4382) links between sequences within Guangdong-Shenzhen and 22.4% (981/4382) links between other provinces. There are 93.3% linkages related to Guangdong-Shenzhen nodes (Table 3 and **Table S3**).

**Table 3.** Links between regions and provinces in the molecular network

Region	Province	BJ	HE	SX	NM	SH	JS	ZJ	AH	FJ	JX	SD	HA	HB	HN	GD	SZ	GX	SC	GZ	YN	SN
North	BJ	31.3	1.8	1.2	0.0	1.2	2.4	0.6	0.6	1.2	0.0	1.8	0.0	0.0	5.4	25.9	16.3	7.8	1.2	0.0	0.0	0.6
	HE	3.8	5.1	0.0	0.0	2.5	0.0	0.0	1.3	0.0	0.0	0.0	0.0	0.0	3.8	57.0	21.5	2.5	0.0	1.3	1.3	0.0
	SX	40.0	0.0	0.0	0.0	0.0	20.0	0.0	0.0	0.0	0.0	0.0	0.0	0.0	40.0	0.0	0.0	0.0	0.0	0.0	0.0	0.0
	NM	0.0	0.0	0.0	9.5	0.0	0.0	9.5	0.0	0.0	0.0	0.0	0.0	0.0	0.0	42.9	19.0	19.0	0.0	0.0	0.0	0.0
East	SH	2.0	2.0	0.0	0.0	8.2	0.0	0.0	1.0	0.0	0.0	0.0	0.0	0.0	5.1	46.9	32.7	2.0	0.0	0.0	0.0	0.0
	JS	6.8	0.0	1.7	0.0	0.0	44.1	0.0	5.1	6.8	0.0	0.0	0.0	0.0	5.1	6.8	23.7	0.0	0.0	0.0	0.0	0.0
	ZJ	6.3	0.0	0.0	12.5	0.0	0.0	0.0	0.0	0.0	0.0	0.0	6.3	0.0	0.0	12.5	25.0	12.5	6.3	6.3	0.0	12.5
	AH	2.8	2.8	0.0	0.0	2.8	8.3	0.0	22.2	0.0	0.0	8.3	0.0	0.0	0.0	13.9	36.1	0.0	0.0	0.0	0.0	2.8
	FJ	20.0	0.0	0.0	0.0	0.0	40.0	0.0	0.0	40.0	0.0	0.0	0.0	0.0	0.0	0.0	0.0	0.0	0.0	0.0	0.0	0.0
	JX	0.0	0.0	0.0	0.0	0.0	0.0	0.0	0.0	0.0	40.0	0.0	0.0	20.0	0.0	40.0	0.0	0.0	0.0	0.0	0.0	0.0
	SD	12.5	0.0	0.0	0.0	0.0	0.0	0.0	12.5	0.0	0.0	66.7	0.0	0.0	0.0	4.2	4.2	0.0	0.0	0.0	0.0	0.0
Centre	HA	0.0	0.0	0.0	0.0	0.0	0.0	3.1	0.0	0.0	0.0	0.0	0.0	6.3	9.4	18.8	53.1	0.0	3.1	0.0	0.0	6.3
	HB	0.0	0.0	0.0	0.0	0.0	0.0	0.0	0.0	0.0	3.9	0.0	7.7	23.1	3.9	34.6	11.5	11.5	0.0	0.0	0.0	0.0
	HN	2.0	0.7	0.4	0.0	1.1	0.7	0.0	0.0	0.0	0.0	0.0	0.7	0.2	18.3	36.9	31.9	2.8	2.0	0.9	1.1	0.2
South	GD	1.1	1.2	0.0	0.2	1.2	0.1	0.1	0.1	0.0	0.1	0.0	0.2	0.2	4.4	53.4	32.4	2.9	0.7	0.6	0.6	0.1
	SZ	0.8	0.5	0.0	0.1	1.0	0.4	0.1	0.4	0.0	0.0	0.0	0.5	0.1	4.4	37.9	49.1	2.1	0.6	0.5	0.6	0.4
	GX	4.5	0.7	0.0	1.4	0.7	0.0	0.7	0.0	0.0	0.0	0.0	0.0	1.0	4.5	38.8	24.6	19.4	0.7	0.7	1.0	0.0
	SC	3.0	0.0	0.0	0.0	0.0	0.0	1.5	0.0	0.0	0.0	0.0	1.5	0.0	13.4	40.3	28.4	3.0	3.0	1.5	1.5	0.0
	GZ	0.0	2.0	0.0	0.0	0.0	0.0	2.0	0.0	0.0	0.0	0.0	0.0	0.0	8.2	46.9	32.7	4.1	2.0	0.0	2.0	0.0
	YN	0.0	1.7	0.0	0.0	0.0	0.0	0.0	0.0	0.0	0.0	0.0	0.0	0.0	8.5	42.4	35.6	5.1	1.7	1.7	3.4	0.0
Northwest	SN	3.5	0.0	0.0	0.0	0.0	0.0	6.9	3.5	0.0	0.0	0.0	6.9	0.0	3.5	13.8	41.4	0.0	0.0	0.0	0.0	20.7

Note: The values in the table are percentages. The 20 provinces with the highest number of links are selected in the table. The darker the colour in the table, the more links there are. The number of links for all provinces is in Table S3**.**

### Analysis of temporal and spatial transmission characteristics of CRF55_01B

**Figure 1.**
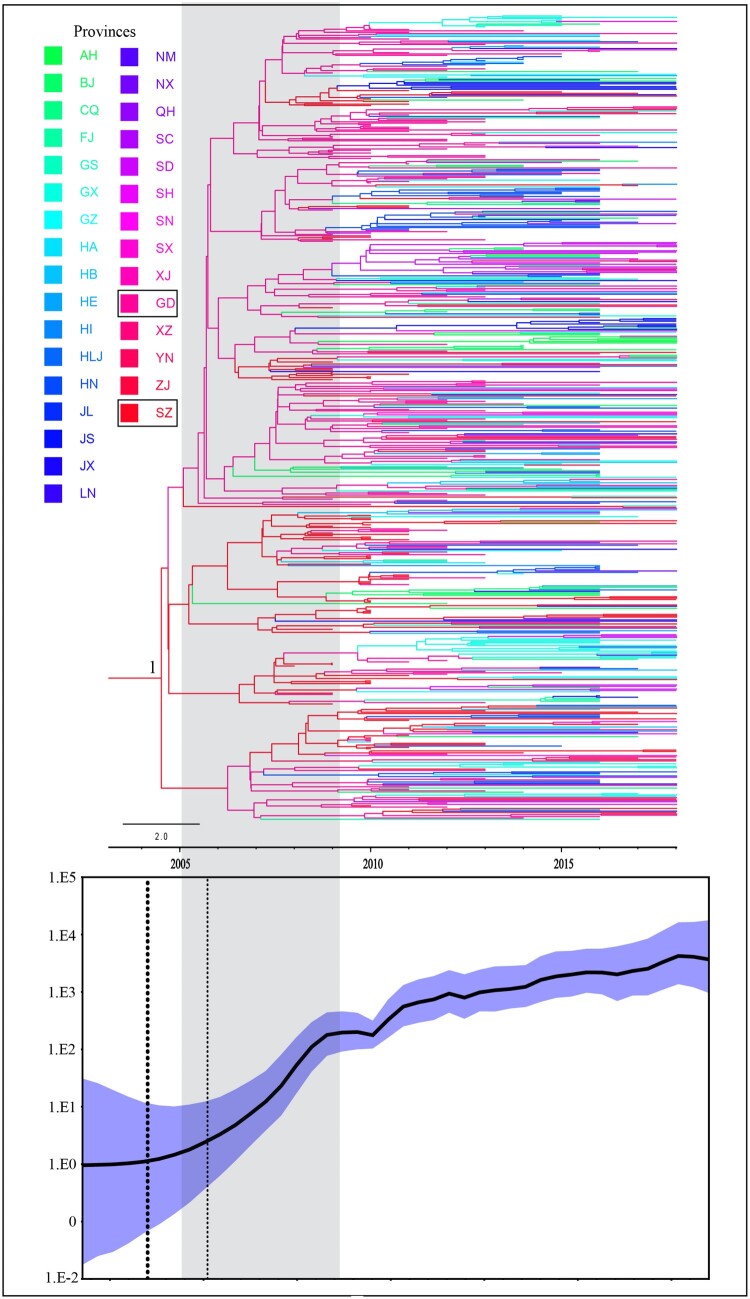
Time-scaled phylogeographic history of CRF55_01B strain. Branch colours represent the most probable province of the parental node of each branch. The MCC trees and Bayesian Skygrid demographic reconstruction share a timeline. The dotted frame shows the period of rapid spread of CRF55_01B strain from 2005 to 2009.

**Figure 2.**
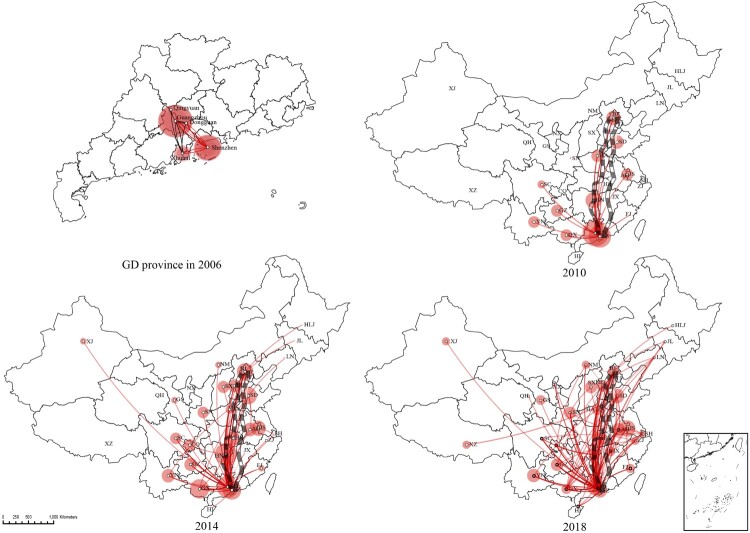
The roadmap of CRF55_01B strain propagating. The lines in the figure show the posterior probability ≥ 0.8. Lines opacity were 0.4. The Beijing-Guangzhou railway is shown in the picture. The red circles indicate the absolute and relative intensity of local CRF55_01B spread.

To reconstruct the epidemic history of CRF55_01B, Bayesian discrete phylogeographic approaches were performed under a Bayesian Skygrid demographic model. The results revealed that CRF55_01B originated in Shenzhen City of Guangdong province (posterior probabilities = 1) early this century. The tMRCA was around 2003.0 (95% HPD interval: 2001.2–2004.6) and the evolutionary rates were around 2.50 (2.21–2.83) × 10^3^ (numbers in parenthesis show the 95% HPD interval). Before it began to spread to other provinces in 2007.9 (95% HPD interval: 2006.8–2009.8), it only diffused within Guangdong province. Time-scaled Bayesian Skygrid demographic shown that CRF55_01B increased exponentially from 2005 to 2009. After 2010, it still maintained rapid and sustained growth and has already spread to the whole country (Figures 1 and 2**)**. Besides, we found that CRF55_01B originated from MSMs and began to spread to heterosexuals around 2007.4 (95% HPD interval: 2006.5–2008.1). It spread to HET in general around 2010 (**Figure S2)**.

**Figure 3.**
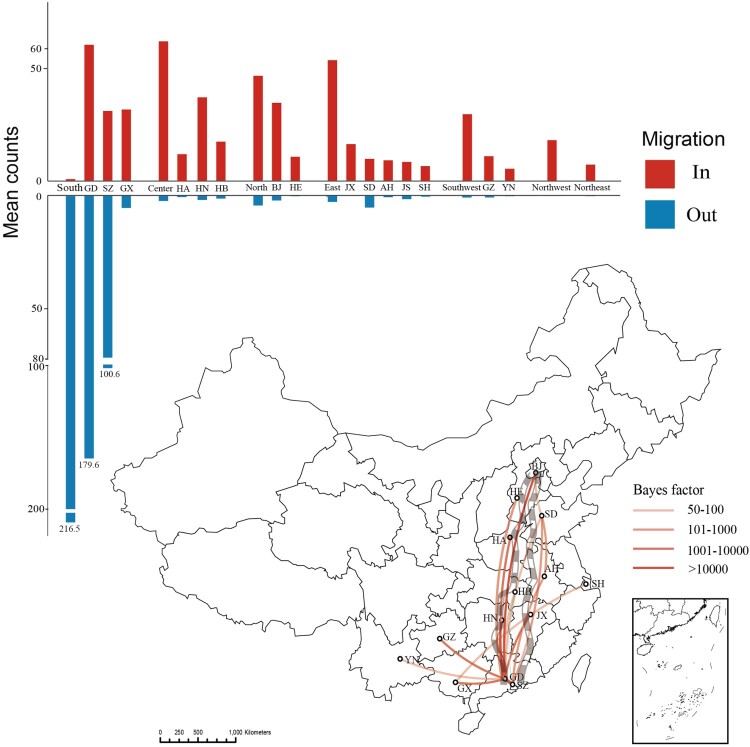
The bar chart shows the input (in) and output (out) of CRF55_01B by regions and provinces. The map shows the provinces where CRF55_01B is most heavily propagated in the BSSVS analysis. The transmission relationships of provinces between BF ≥ 50 and posterior probability ≥ 0.8 are shown in the figure. Mean counts are the average counts from A to B calculated by the MCC trees. South China has very little inputs in the bar, but GD and SZ have a lot. This is because south China mean counts from other areas. GD and SZ import more because they spread more to each other.

To describe and quantify CRF55_01B movement, we next performed the BSSVS analysis. We found that transitions between regions with the highest support were from South to Centre (mean counts = 65.06, BF > 10000, posterior probability = 1), from South to East (mean counts = 55.81, BF > 10000, posterior probability = 1), and from South to North (mean counts = 46.61, BF > 10000, posterior probability = 1). Transitions between provinces with the highest support were from Shenzhen to Guangdong (mean counts = 62.21, BF > 10000, posterior probability = 1), from Guangdong to Hunan (mean counts = 36.20, BF > 10000, posterior probability = 1), from Guangdong to Shenzhen (mean counts = 30.44, BF > 10000, posterior probability = 1). Guangdong, Shenzhen, Hunan, Beijing, Guangxi, Hubei, Jiangxi, Guizhou, Hebei, Anhui, Shanghai, Shandong, Henan, and Yunnan (BF ≥ 50, posterior probability ≥ 0.8) were the key provinces of CRF55_01B transmission (Figure 3 and **Table S4–5**).

**Figure 4.**
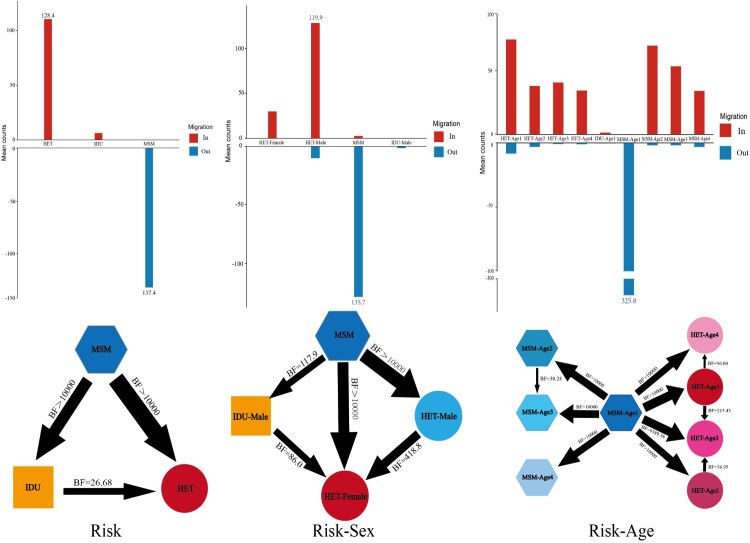
The bar chart shows the input (in) and output (out) of CRF55_01B by Risks, Risk-Sexs and Risk-Ages. The transmission relationships among different groups are depicted in the figure. The transmission relationships between BF ≥ 50 and posterior probability ≥ 0.8 were shown in the figure. The thickness of the arrows in the figure indicates the magnitude of BF and the posterior probability.

Transitions between Risks with the highest support was from MSMs to heterosexuals (mean counts = 128.4, BF > 10000, posterior probability = 1). Transitions between Risk-Sexs with the highest support was from MSMs to males in heterosexuals (mean counts = 119.93, BF > 10000, posterior probability = 1). Transitions between Risk-Ages with the highest support were from MSMs aged 16–29 to heterosexuals aged 16–29 (mean counts = 72.31, BF > 10000, posterior probability = 1), MSMs aged 16–29 to MSMs aged 30–39 (mean counts = 68.38, BF > 10000, posterior probability = 1) (Figure 4 and **Table S6–8**.)

### Phylogenetic and geographic analysis of CRF55_01B

To statistically quantify the degree of diversification achieved by CRF55_01B, a BaTS was carried out. Phylogeny showed evidence of geographic association of CRF55_01B assessed by AI and PS (*P* = 0.00) in 31 provinces. Besides, the analysis of the MC index showed that the association was statistically supported (*P* < 0.05) for Guangdong, Shenzhen, Jiangsu, Guangxi, Shandong, Hunan, Yunnan, Beijing, Anhui, Shaanxi, Hubei, Shanghai, Guizhou, Xinjiang, Zhejiang, Tibet, Jiangxi, Hebei, Chongqing, Fujian. This result suggests at least some historical circulation of CRF55_01B in these provinces (**Table S9**).

### Correlation between the dissemination of CRF55_01B and the Beijing-Guangzhou/Beijing-Kowloon railways

**Figure 5.**
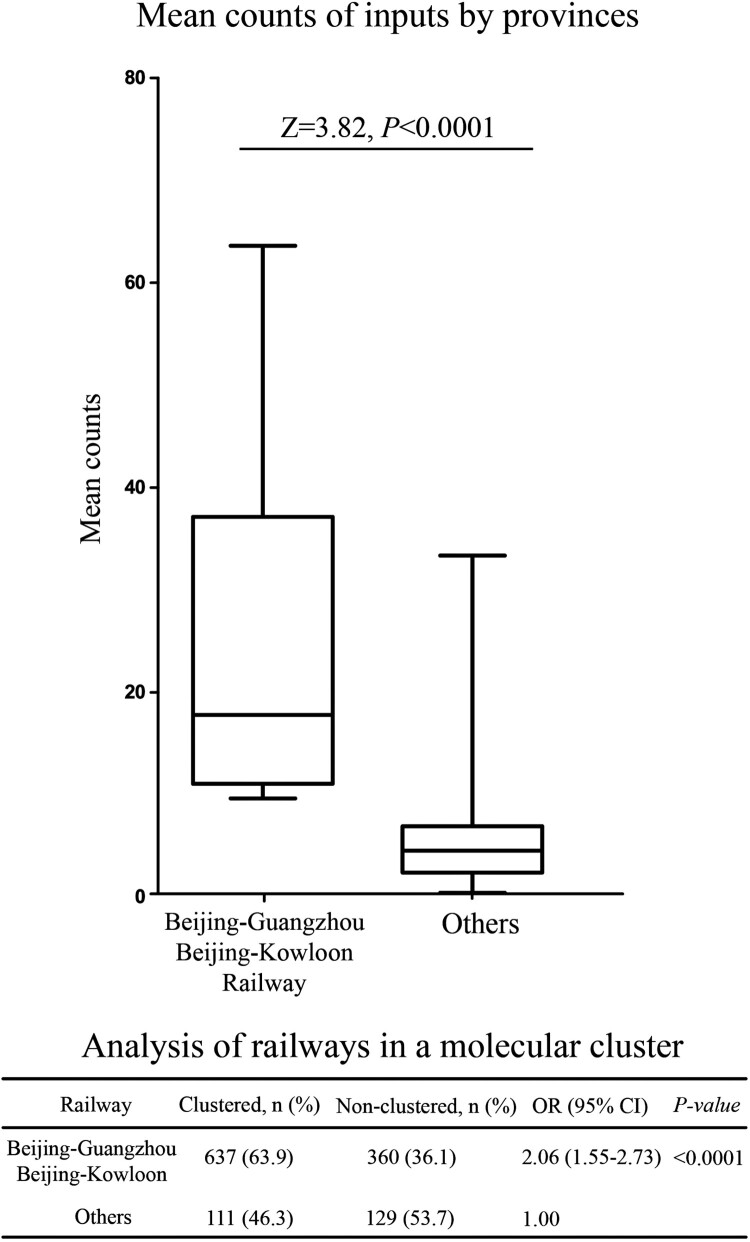
Correlation between the dissemination of CRF55_01B and the Beijing-Guangzhou/Beijing-Kowloon railways.

The propagation roadmap of CRF55_01B (Figure 2) showed that the Guangdong-Beijing line was particularly significant. Therefore, we think of the Beijing-Guangzhou and Beijing-Kowloon railways. To verify the hypothesis that CRF55_01B spread along with the development of the Beijing-Guangzhou and Beijing-Kowloon railways, we added the provinces’ comparative analysis on the Beijing-Guangzhou and Beijing-Kowloon railways belonging to the molecular cluster and those not belonging to the molecular cluster. CRF55_01B was found to be more easily accessible to the molecular network in the provinces on the Beijing-Guangzhou and Beijing-Kowloon railways (*P* < 0.0001). The mean counts of CRF55_01B import provinces were compared between located on the Beijing-Guangzhou and Beijing-Kowloon railways and located on others. The results demonstrated the provinces with more CRF55_01B inputs were mostly located on the Beijing-Guangzhou and Beijing-Kowloon railways (*P* < 0.0001) (Figure 5).

## Discussion

Using more representative national sequences (1237 sequences, 2007–2018), our study systematically analyzed the origin and spread of CRF55_01B and reconstructed its epidemic history. The tMRCA of CRF55_01B was around 2003.0 (95% HPD interval: 2001.2–2004.6). The estimate was similar to previous studies [5–7,28], but the 95% HPD interval was narrower, possibly caused by our more complete data set.

This study proved that Guangdong-Shenzhen in South China was the source of infection for the rapid spread of CRF55_01B to other provinces in China. CRF55_01B first spread to other cities in Guangdong after its origin in Shenzhen, gradually spread from Guangdong to other provinces around 2007, and then spread to the whole country after 2010.

After reconstructing the epidemic history of CRF55_01B, we found that the spread and diffusion of CRF55_01B closely related to traffic development. In 2007, the sixth-speed increase of China railway (including the Beijing-Guangzhou and Beijing-Kowloon railways) marked China's entry into the ranks of the world's advanced railways. Guangdong-Shenzhen is a major economic province in China, with GDP ranking among the top in the country and rapid development. After the acceleration of the Beijing-Guangzhou railway in 2007, passengers of the Beijing-Guangzhou railway increased significantly. The Beijing-Guangzhou railway is the busiest railway line in China and the most important north–south railway transportation artery in China, running through Beijing, Hebei, Henan, Hubei, Hunan, and Guangdong provinces. It is the railway with the most connections through provincial capitals and other railways in China. Since 2010, the completion of high-speed rail networks in the Yangtze River Delta, Pearl River Delta, and other regions, as well as the Beijing-Guangzhou high-speed railway, the world's longest in operation, has brought Guangdong closer to other provinces. The Beijing-Kowloon railway, which connects not only the north and south but also the central and eastern regions, has become a busy railway in China. It passes through Beijing, Hebei, Shangdong, Anhui, Henan, Hubei, Jiangxi, Guangdong, and Shenzhen. The southern end of the Beijing-Guangzhou Railway and the Beijing-Kowloon Railway is Shenzhen. The provinces where CRF55_01B mainly spread happened to be the provinces through which the Beijing-Guangzhou and Beijing-Kowloon railways passed. Therefore, CRF55_01B may spread rapidly along with the rapid development of the Beijing-Guangzhou and Beijing-Kowloon railways.

CRF55_01B, as a strain of MSM origin, is more likely to spread between large cities and across provinces like other MSM strains [29,30]. At the same time, we have also noticed that CRF55_01B is gradually spreading in railway lines related to the Beijing-Guangzhou and Beijing-Kowloon railways, such as Guangxi, Yunnan, Guizhou, and other places in the west of the railway, and Fujian, Jiangsu, Zhejiang, Shanghai, and other places in the east of the railway.

After CRF55_01B spread to other provinces, the occurrence of CRF55_01B in each province also varies. This study found that CRF55_01B had some historical circulation in 19 provinces (**Table S9)**. Compared with other studies [7,12], this study obtained more information about the spread of CRF55_01B in other provinces.

Although CRF55_01 was first detected in MSMs, it quickly spread to heterosexuals. We found that heterosexuals showed an increasing trend in three periods (*P* < 0.0001), Table 2. It is worth noting that this is probably due to the greatly reduced “Unknown” population during 2016–2018. In this study, both the molecular network analysis and Bayesian correlation analysis showed a very close relationship between MSMs and heterosexuals. CRF55_01B has an obvious trend of spread from MSMs to heterosexuals, mainly from young MSMs to young heterosexuals males (Figure 4**)**. The transmission route of HIV-1 in China changed significantly from blood transfusion transmission to sexual transmission between 2007 and 2009 [31,32]. During this period, CRF55_01B also experienced a transmission transition from MSMs to heterosexuals. Therefore, CRF55_01B is still mainly concentrated in MSMs at present, but the number of heterosexually transmitted infections is increasing. This is consistent with the MSMs transmission mode in China, which is transmitted among young people, and males in heterosexuals play a crucial role in the transmission. This mode of transmission may be related to the fact that there are many non-disclosed MSMs in China. Because of social factors, Chinese MSMs are partially self-reported as heterosexuals and bisexuals, and they may have both homosexual and heterosexual behaviours at the same time. Other studies have also proved the existence of such a transmission relationship among Chinese MSMs [33,34].

CRF55_01B is a relatively “young” HIV strain, but it is not spreading at all slowly. From 2013 to 2018, CRF55_01B has become the fifth largest strain China’s the HIV-1 composition ratio within 5 years. It has been found in all China provinces and formed transmission clusters in more than half of the provinces. The transmission of CRF55_01B may be related to the development of transportation and technology. CRF55_01B was not found in the Los Alamos National Laboratories (LANL) HIV sequence database in any other country except China. It is also an HIV strain circulating in China. The CRF55_01B is transmitted from MSMs to heterosexuals, so it is necessary to be aware of the reverse transmission of heterosexuals into MSMs. Besides, other studies have shown that CRF55_01B may have a higher transmission risk than CRF01_AE and CRF07_BC [35]. Therefore, the prevention and control of CRF55_01B need to be concerned. As a representative of newly discovered strains of HIV-1 in China, especially MSM strain, its transmission characteristics just reflect the epidemic status of MSM strains in China. The analysis of CRF55_01B suggests that if we detect the spread of MSMs in time through molecular monitoring in the early stage of the epidemic, it will help us control the epidemic early and prevent its spread, which is of great significance to China's national prevention and control of HIV-1. Additionally, this strain originated in developed cities and had convenient transportation, which closely related to the development of China's railways. It is suggested that the spread of HIV-1 closely related to socio-economic development and more attention was needed.

## Supplementary Material

Supplementary_materials-clean.doc
