# Supplementary material for: The prevalence of CRF55_01B among HIV-1 strain and its connection with traffic development in China
Source: Emerg Microbes Infect. 2021 Feb 18;10(1):256–65. doi: 10.1080/22221751.2021.1884004 (PMC7894451; doi:10.1080/22221751.2021.1884004)
Supplement: Supplementary_materials-clean.doc [file TEMI_A_1884004_SM2157.doc]

Supplementary materials for

**The prevalence of CRF55_01B among HIV-1 strain and its connection with traffic development in China**

Mengze Gan^a^, Shan Zheng^a^, Jingjing Hao^a^, Yuhua Ruan^a^, Lingjie Liao^a^, Yiming Shao^a^, Yi Feng^a*^ and Hui Xing^a*^

*^a^State Key Laboratory of Infectious Disease Prevention and Control, National Center for AIDS/STD Control and Prevention (NCAIDS), Chinese Center for Disease Control and Prevention (China CDC), Collaborative Innovation Center for Diagnosis and Treatment of Infectious Diseases, Beijing 102206, China*

***Corresponding author:** Yi Feng, [fengyi@chinaaids.cn](mailto:fengyi@chinaaids.cn), Hui Xing, [xingh@chinaaids.cn](mailto:xingh@chinaaids.cn), Division of Virology and Immunology, National Center for AIDS/STD Control and Prevention (NCAIDS), Chinese Center for Disease Control and Prevention (China-CDC), Room 509, No.155 Changbai Road, Changping District, Beijing 102206, People's Republic of China

**Table S1.** Distribution of sequences by regions and provinces

|  | Data set A | Data set B |
| --- | --- | --- |
| Overall | 1237 (100.0) | 593 (47.9) |
| North |  |  |
| BJ | 75 (6.1) | 39 (6.6) |
| TJ | 3 (0.2) | 0 (0.0) |
| HE | 21 (1.7) | 13 (2.2) |
| SX | 5 (0.4) | 3 (0.5) |
| NM | 5 (0.4) | 5 (0.8) |
| Northeast |  |  |
| LN | 4 (0.3) | 4 (0.7) |
| JL | 3 (0.2) | 3 (0.5) |
| HLJ | 3 (0.2) | 1 (0.2) |
| East |  |  |
| SH | 32 (2.6) | 6 (1.0) |
| JS | 24 (1.9) | 19 (3.2) |
| ZJ | 11 (0.9) | 4 (0.7) |
| AH | 19 (1.5) | 13 (2.2) |
| FJ | 6 (0.5) | 5 (0.8) |
| JX | 16 (1.3) | 15 (2.5) |
| SD | 27 (2.2) | 26 (4.4) |
| Center |  |  |
| HA | 13 (1.1) | 9 (1.5) |
| HB | 22 (1.8) | 17 (2.9) |
| HN | 68 (5.5) | 59 (9.9) |
| South |  |  |
| GD | 454 (36.7) | 144 (24.3) |
| SZ | 283 (22.9) | 106 (17.9) |
| GX | 71 (5.7) | 44 (7.4) |
| HI | 5 (0.4) | 4 (0.7) |
| Southwest |  |  |
| CQ | 10 (0.8) | 7 (1.2) |
| SC | 7 (0.6) | 7 (1.2) |
| GZ | 12 (1.0) | 11 (1.9) |
| YN | 11 (0.9) | 9 (1.5) |
| XZ | 2 (0.2) | 2 (0.3) |
| Northwest |  |  |
| SN | 16 (1.3) | 9 (1.5) |
| GS | 3 (0.2) | 3 (0.5) |
| QH | 1 (0.1) | 1 (0.2) |
| NX | 3 (0.2) | 3 (0.5) |
| XJ | 2 (0.2) | 2 (0.3) |

**Table S2.** Demographic characteristics of the study population according to whether they belong to a molecular cluster

|  | Overall (%) | Non-clustered (%) | Clustered (%) | *P*-value^*^ |
| --- | --- | --- | --- | --- |
| Overall | 1237 (100.0) | 489 (39.5) | 748 (60.5) |  |
| Sex |  |  |  | 0.097 |
| Male | 680 (55.0) | 277 (56.6) | 403 (53.9) |  |
| Female | 40 (3.2) | 21 (4.3) | 19 (2.5) |  |
| Unknown | 517 (41.8) | 191 (39.1) | 326 (43.6) |  |
| Age (years) |  |  |  | <0.001 |
| 16~29 | 231 (18.7) | 102 (20.9) | 129 (17.2) |  |
| 30~39 | 98 (7.9) | 56 (11.5) | 42 (5.6) |  |
| 40~49 | 86 (7.0) | 50 (10.2) | 36 (4.8) |  |
| ≥50 | 55 (4.4) | 33 (6.7) | 22 (2.9) |  |
| Unknown | 767 (62.0) | 248 (50.7) | 519 (69.4) |  |
| Risk |  |  |  | 0.003 |
| MSM | 520 (42.0) | 192 (39.3) | 328 (43.9) |  |
| HET | 214 (17.3) | 109 (22.3) | 105 (14.0) |  |
| IDU | 10 (0.8) | 4 (0.8) | 6 (0.8) |  |
| Unknown | 493 (39.9) | 184 (37.6) | 309 (41.3) |  |
| Sampling year |  |  |  | <0.05 |
| 2007-2012 | 427 (34.5) | 82 (16.8) | 345 (46.1) |  |
| 2013-2015 | 493 (39.9) | 206 (42.1) | 287 (38.4) |  |
| 2016-2018 | 317 (25.6) | 201 (41.1) | 116 (15.5) |  |
| Region |  |  |  | <0.001 |
| Center | 103 (8.3) | 46 (9.4) | 57 (7.6) |  |
| East | 135 (10.9) | 68 (13.9) | 67 (9.0) |  |
| North | 109 (8.8) | 42 (8.6) | 67 (9.0) |  |
| Northeast | 10 (0.8) | 8 (1.6) | 2 (0.3) |  |
| Northwest | 25 (2.0) | 15 (3.1) | 10 (1.3) |  |
| South | 813 (65.7) | 285 (58.3) | 528 (70.5) |  |
| Southwest | 42 (3.4) | 25 (5.1) | 17 (2.3) |  |
| Province |  |  |  | <0.001 |
| AH | 19 (1.5) | 7 (1.4) | 12 (1.6) |  |
| BJ | 75 (6.1) | 25 (5.1) | 50 (6.7) |  |
| CQ | 10 (0.8) | 6 (1.2) | 4 (0.5) |  |
| FJ | 6 (0.5) | 3 (0.6) | 3 (0.4) |  |
| GD | 454 (36.7) | 168 (34.4) | 286 (38.2) |  |
| GS | 3 (0.2) | 2 (0.4) | 1 (0.1) |  |
| GX | 71 (5.7) | 33 (6.7) | 38 (5.1) |  |
| GZ | 12 (1.0) | 9 (1.8) | 3 (0.4) |  |
| HA | 13 (1.1) | 7 (1.4) | 6 (0.8) |  |
| HB | 22 (1.8) | 13 (2.7) | 9 (1.2) |  |
| HE | 21 (1.7) | 8 (1.6) | 13 (1.7) |  |
| HI | 5 (0.4) | 3 (0.6) | 2 (0.3) |  |
| HLJ | 3 (0.2) | 2 (0.4) | 1 (0.1) |  |
| HN | 68 (5.5) | 26 (5.3) | 42 (5.6) |  |
| JL | 3 (0.2) | 3 (0.6) | 0 (0.0) |  |
| JS | 24 (1.9) | 9 (1.8) | 15 (2.0) |  |
| JX | 16 (1.3) | 12 (2.5) | 4 (0.5) |  |
| LN | 4 (0.3) | 3 (0.6) | 1 (0.1) |  |
| NM | 5 (0.4) | 3 (0.6) | 2 (0.3) |  |
| NX | 3 (0.2) | 2 (0.4) | 1 (0.1) |  |
| QH | 1 (0.1) | 1 (0.2) | 0 (0.0) |  |
| SC | 7 (0.6) | 3 (0.6) | 4 (0.5) |  |
| SD | 27 (2.2) | 13 (2.7) | 14 (1.9) |  |
| SH | 32 (2.6) | 17 (3.5) | 15 (2.0) |  |
| SN | 16 (1.3) | 8 (1.6) | 8 (1.1) |  |
| SX | 5 (0.4) | 3 (0.6) | 2 (0.3) |  |
| SZ | 283 (22.9) | 81 (16.6) | 202 (27.0) |  |
| TJ | 3 (0.2) | 3 (0.6) | 0 (0.0) |  |
| XJ | 2 (0.2) | 2 (0.4) | 0 (0.0) |  |
| XZ | 2 (0.2) | 0 (0.0) | 2 (0.3) |  |
| YN | 11 (0.9) | 7 (1.4) | 4 (0.5) |  |
| ZJ | 11 (0.9) | 7 (1.4) | 4 (0.5) |  |

*****: If the theoretical number of cells was less than 5, Fisher test was used.

**Table S3.** links between regions and provinces in the molecular network

| Region | Province | BJ | HE | SX | NM | HLJ | LN | SH | JS | ZJ | AH | FJ | JX | SD | HA | HB | HN | GD | SZ | GX | HI | CQ | SC | GZ | YN | XZ | SN | GS | NX |
| --- | --- | --- | --- | --- | --- | --- | --- | --- | --- | --- | --- | --- | --- | --- | --- | --- | --- | --- | --- | --- | --- | --- | --- | --- | --- | --- | --- | --- | --- |
| North | BJ | 52 | 3 | 2 | 0 | 1 | 0 | 2 | 4 | 1 | 1 | 2 | 0 | 3 | 0 | 0 | 9 | 43 | 27 | 13 | 0 | 0 | 2 | 0 | 0 | 0 | 1 | 0 | 0 |
|  | HE | 3 | 4 | 0 | 0 | 0 | 0 | 2 | 0 | 0 | 1 | 0 | 0 | 0 | 0 | 0 | 3 | 45 | 17 | 2 | 0 | 0 | 0 | 1 | 1 | 0 | 0 | 0 | 0 |
|  | SX | 2 | 0 | 0 | 0 | 0 | 0 | 0 | 1 | 0 | 0 | 0 | 0 | 0 | 0 | 0 | 2 | 0 | 0 | 0 | 0 | 0 | 0 | 0 | 0 | 0 | 0 | 0 | 0 |
|  | NM | 0 | 0 | 0 | 2 | 0 | 0 | 0 | 0 | 2 | 0 | 0 | 0 | 0 | 0 | 0 | 0 | 9 | 4 | 4 | 0 | 0 | 0 | 0 | 0 | 0 | 0 | 0 | 0 |
| Northeast | HLJ | 1 | 0 | 0 | 0 | 0 | 0 | 0 | 0 | 0 | 0 | 0 | 0 | 0 | 0 | 0 | 0 | 0 | 0 | 0 | 0 | 0 | 0 | 0 | 0 | 0 | 0 | 0 | 0 |
|  | LN | 0 | 0 | 0 | 0 | 0 | 0 | 0 | 0 | 0 | 0 | 0 | 0 | 0 | 0 | 1 | 0 | 0 | 0 | 1 | 0 | 0 | 0 | 0 | 0 | 0 | 0 | 0 | 0 |
| East | SH | 2 | 2 | 0 | 0 | 0 | 0 | 8 | 0 | 0 | 1 | 0 | 0 | 0 | 0 | 0 | 5 | 46 | 32 | 2 | 0 | 0 | 0 | 0 | 0 | 0 | 0 | 0 | 0 |
|  | JS | 4 | 0 | 1 | 0 | 0 | 0 | 0 | 26 | 0 | 3 | 4 | 0 | 0 | 0 | 0 | 3 | 4 | 14 | 0 | 0 | 0 | 0 | 0 | 0 | 0 | 0 | 0 | 0 |
|  | ZJ | 1 | 0 | 0 | 2 | 0 | 0 | 0 | 0 | 0 | 0 | 0 | 0 | 0 | 1 | 0 | 0 | 2 | 4 | 2 | 0 | 0 | 1 | 1 | 0 | 0 | 2 | 0 | 0 |
|  | AH | 1 | 1 | 0 | 0 | 0 | 0 | 1 | 3 | 0 | 8 | 0 | 0 | 3 | 0 | 0 | 0 | 5 | 13 | 0 | 0 | 0 | 0 | 0 | 0 | 0 | 1 | 0 | 0 |
|  | FJ | 2 | 0 | 0 | 0 | 0 | 0 | 0 | 4 | 0 | 0 | 4 | 0 | 0 | 0 | 0 | 0 | 0 | 0 | 0 | 0 | 0 | 0 | 0 | 0 | 0 | 0 | 0 | 0 |
|  | JX | 0 | 0 | 0 | 0 | 0 | 0 | 0 | 0 | 0 | 0 | 0 | 2 | 0 | 0 | 1 | 0 | 2 | 0 | 0 | 0 | 0 | 0 | 0 | 0 | 0 | 0 | 0 | 0 |
|  | SD | 3 | 0 | 0 | 0 | 0 | 0 | 0 | 0 | 0 | 3 | 0 | 0 | 16 | 0 | 0 | 0 | 1 | 1 | 0 | 0 | 0 | 0 | 0 | 0 | 0 | 0 | 0 | 0 |
| Center | HA | 0 | 0 | 0 | 0 | 0 | 0 | 0 | 0 | 1 | 0 | 0 | 0 | 0 | 0 | 2 | 3 | 6 | 17 | 0 | 0 | 0 | 1 | 0 | 0 | 0 | 2 | 0 | 0 |
|  | HB | 0 | 0 | 0 | 0 | 0 | 1 | 0 | 0 | 0 | 0 | 0 | 1 | 0 | 2 | 6 | 1 | 9 | 3 | 3 | 0 | 0 | 0 | 0 | 0 | 0 | 0 | 0 | 0 |
|  | HN | 9 | 3 | 2 | 0 | 0 | 0 | 5 | 3 | 0 | 0 | 0 | 0 | 0 | 3 | 1 | 84 | 169 | 146 | 13 | 0 | 1 | 9 | 4 | 5 | 0 | 1 | 0 | 0 |
| South | GD | 43 | 45 | 0 | 9 | 0 | 0 | 46 | 4 | 2 | 5 | 0 | 2 | 1 | 6 | 9 | 169 | 2070 | 1257 | 112 | 1 | 16 | 27 | 23 | 25 | 0 | 4 | 0 | 0 |
|  | SZ | 27 | 17 | 0 | 4 | 0 | 0 | 32 | 14 | 4 | 13 | 0 | 0 | 1 | 17 | 3 | 146 | 1257 | 1628 | 71 | 1 | 8 | 19 | 16 | 21 | 0 | 12 | 2 | 4 |
|  | GX | 13 | 2 | 0 | 4 | 0 | 1 | 2 | 0 | 2 | 0 | 0 | 0 | 0 | 0 | 3 | 13 | 112 | 71 | 56 | 0 | 2 | 2 | 2 | 3 | 0 | 0 | 0 | 1 |
|  | HI | 0 | 0 | 0 | 0 | 0 | 0 | 0 | 0 | 0 | 0 | 0 | 0 | 0 | 0 | 0 | 0 | 1 | 1 | 0 | 0 | 0 | 0 | 0 | 0 | 0 | 0 | 0 | 0 |
| Southwest | CQ | 0 | 0 | 0 | 0 | 0 | 0 | 0 | 0 | 0 | 0 | 0 | 0 | 0 | 0 | 0 | 1 | 16 | 8 | 2 | 0 | 0 | 2 | 0 | 0 | 0 | 0 | 0 | 0 |
|  | SC | 2 | 0 | 0 | 0 | 0 | 0 | 0 | 0 | 1 | 0 | 0 | 0 | 0 | 1 | 0 | 9 | 27 | 19 | 2 | 0 | 2 | 2 | 1 | 1 | 0 | 0 | 0 | 0 |
|  | GZ | 0 | 1 | 0 | 0 | 0 | 0 | 0 | 0 | 1 | 0 | 0 | 0 | 0 | 0 | 0 | 4 | 23 | 16 | 2 | 0 | 0 | 1 | 0 | 1 | 0 | 0 | 0 | 0 |
|  | YN | 0 | 1 | 0 | 0 | 0 | 0 | 0 | 0 | 0 | 0 | 0 | 0 | 0 | 0 | 0 | 5 | 25 | 21 | 3 | 0 | 0 | 1 | 1 | 2 | 0 | 0 | 0 | 0 |
|  | XZ | 0 | 0 | 0 | 0 | 0 | 0 | 0 | 0 | 0 | 0 | 0 | 0 | 0 | 0 | 0 | 0 | 0 | 0 | 0 | 0 | 0 | 0 | 0 | 0 | 2 | 0 | 0 | 0 |
| Northwest | SN | 1 | 0 | 0 | 0 | 0 | 0 | 0 | 0 | 2 | 1 | 0 | 0 | 0 | 2 | 0 | 1 | 4 | 12 | 0 | 0 | 0 | 0 | 0 | 0 | 0 | 6 | 0 | 0 |
|  | GS | 0 | 0 | 0 | 0 | 0 | 0 | 0 | 0 | 0 | 0 | 0 | 0 | 0 | 0 | 0 | 0 | 0 | 2 | 0 | 0 | 0 | 0 | 0 | 0 | 0 | 0 | 0 | 0 |
|  | NX | 0 | 0 | 0 | 0 | 0 | 0 | 0 | 0 | 0 | 0 | 0 | 0 | 0 | 0 | 0 | 0 | 0 | 4 | 1 | 0 | 0 | 0 | 0 | 0 | 0 | 0 | 0 | 0 |

Note: The darker the color in the table, the more links there are.

**Table S4. Results of BSSVS in the regions**

| From | To | Mean counts | Bayes Factor | Posterior probability^*^ |
| --- | --- | --- | --- | --- |
| South | Center | 65.06 | ＞10000 | 1.00 |
| South | East | 55.81 | ＞10000 | 1.00 |
| South | North | 46.61 | ＞10000 | 1.00 |
| South | Southwest | 26.47 | ＞10000 | 1.00 |
| South | Northwest | 18.71 | ＞10000 | 1.00 |
| North | Northeast | 3.23 | 295.06 | 0.98 |
| Center | Southwest | 2.02 | 39.61 | 0.88 |
| East | Southwest | 2.67 | 22.02 | 0.81 |

*: The transmission relationships with posterior probability≥0.8 were selected.

**Table S5. Results of BSSVS in the provinces**

| From | To | Mean counts | Bayes Factor | Posterior probability^*^ |
| --- | --- | --- | --- | --- |
| SZ | GD | 62.21 | ＞10000 | 1.00 |
| GD | HN | 36.20 | ＞10000 | 1.00 |
| GD | SZ | 30.44 | ＞10000 | 1.00 |
| GD | BJ | 24.87 | ＞10000 | 1.00 |
| GD | GX | 25.18 | 3511.95 | 1.00 |
| GD | JX | 15.98 | 1857.37 | 0.99 |
| GD | HA | 11.45 | 1277.46 | 0.99 |
| GD | GZ | 11.34 | 1260.04 | 0.99 |
| GD | HE | 8.43 | 319.02 | 0.96 |
| SZ | AH | 5.66 | 172.42 | 0.93 |
| SD | AH | 2.33 | 139.36 | 0.92 |
| SD | SZ | 1.56 | 125.84 | 0.91 |
| SD | BJ | 2.32 | 94.50 | 0.88 |
| GD | SD | 7.98 | 83.49 | 0.87 |
| GX | SH | 1.51 | 71.78 | 0.85 |
| GD | HB | 12.35 | 62.44 | 0.84 |
| GD | YN | 4.55 | 58.24 | 0.83 |

*: The transmission relationships with posterior probability≥0.8 were selected.

**Table S6. Results of BSSVS in the risk**

| From | To | Mean counts | Bayes Factor | Posterior probability^*^ |
| --- | --- | --- | --- | --- |
| MSM | HET | 128.36 | >10000 | 1.00 |
| MSM | IDU | 9.07 | >10000 | 1.00 |
| IDU | HET | 0.03 | 26.68 | 0.96 |

*: The transmission relationships with posterior probability≥0.8 were selected. Risks are classified into MSM, heterosexual (HET), and injecting drug users (IDU).

**Table S7. Results of BSSVS in the Risk-Sex**

| From | To | Mean counts | Bayes Factor | Posterior probability^*^ |
| --- | --- | --- | --- | --- |
| MSM | HET-Male | 119.93 | >10000 | 1.00 |
| MSM | HET-Female | 15.82 | >10000 | 1.00 |
| HET-Male | HET-Female | 8.79 | 418.85 | 0.99 |
| MSM | IDU-Male | 2.24 | 117.85 | 0.98 |
| IDU-Male | HET--Female | 1.28 | 85.99 | 0.97 |

*: The transmission relationships with posterior probability≥0.8 were selected. Risk-Sexs are classified into HET-Female (females in heterosexuals), HET-Male (males in heterosexuals), IDU-Male (males in injecting drug users), and MSM.

**Table S8. Results of BSSVS in the Risk-Age**

| From | To | Mean counts | Bayes Factor | Posterior probability^*^ |
| --- | --- | --- | --- | --- |
| MSM-Age1 | HET-Age1 | 72.31 | ＞10000 | 1.00 |
| MSM-Age1 | MSM-Age2 | 68.38 | ＞10000 | 1.00 |
| MSM-Age1 | MSM-Age3 | 51.22 | ＞10000 | 1.00 |
| MSM-Age1 | HET-Age2 | 35.75 | ＞10000 | 1.00 |
| MSM-Age1 | MSM-Age4 | 33.62 | ＞10000 | 1.00 |
| MSM-Age1 | HET-Age4 | 32.51 | ＞10000 | 1.00 |
| MSM-Age1 | HET-Age3 | 31.71 | 9389.58 | 1.00 |
| HET-Age1 | HET-Age3 | 5.62 | 215.43 | 0.97 |
| HET-Age1 | HET-Age4 | 1.58 | 56.04 | 0.89 |
| HET-Age2 | HET-Age3 | 2.71 | 36.95 | 0.84 |
| MSM-Age2 | MSM-Age3 | 1.29 | 30.24 | 0.81 |

*: The transmission relationships with posterior probability≥0.8 were selected. Risk-Ages are classified into HET-Age1 (16-29 years old), HET-Age2 (30-39 years old), HET-Age3 (40-49 years old), HET-Age4 (≥50 years old), IDU-Age1 (16-29 years old), MSM-Age1 (16-29 years old), MSM-Age2 (30-39 years old), MSM-Age3 (40-49 years old) and MSM-Age4 (≥50 years old).

**Table S9. Results of Bayesian Tip-association Significance Testing (BaTS) in provinces**

| Statistic | observed mean (95% CI) | null mean (95% CI) | *P*-value |
| --- | --- | --- | --- |
| **AI** | **63.4 (60.8-65.9)** | **111.4 (108.0-114.3)** | **0.00** |
| **PS** | **470.9 (463.0-479.0)** | **702.4 (693.2-709.9)** | **0.00** |
| **MC(GD)** | **12.1 (8.0-16.0)** | **4.9 (3.9-6.3)** | **0.01** |
| **MC(SZ)** | **9.0 (8.0-11.0)** | **3.1 (2.4-4.0)** | **0.01** |
| **MC(JS)** | **7.8 (5.0-8.0)** | **1.1 (1.0-1.7)** | **0.01** |
| **MC(GX)** | **7.2 (6.0-8.0)** | **1.8 (1.1-2.2)** | **0.01** |
| **MC(SD)** | **5.2 (5.0-7.0)** | **1.2 (1.0-2.0)** | **0.01** |
| **MC(HN)** | **5.0 (4.0-6.0)** | **1.7 (1.1-2.1)** | **0.01** |
| **MC(YN)** | **4.0 (4.0-4.0)** | **1.0 (1.0-1.2)** | **0.01** |
| **MC(BJ)** | **3.9 (3.0-5.0)** | **1.8 (1.2-2.1)** | **0.01** |
| **MC(AH)** | **3.5 (2.0-4.0)** | **1.0 (1.0-1.2)** | **0.01** |
| **MC(SN)** | **3.5 (2.0-5.0)** | **1.0 (1.0-1.3)** | **0.01** |
| **MC(HB)** | **3.2 (2.0-5.0)** | **1.1 (1.0-1.4)** | **0.01** |
| **MC(SH)** | **2.9 (2.0-3.0)** | **1.2 (1.0-1.9)** | **0.01** |
| **MC(GZ)** | **2.0 (2.0-2.0)** | **1.0 (1.0-1.1)** | **0.01** |
| **MC(XJ)** | **2.0 (2.0-2.0)** | **1.0 (1.0-1.0)** | **0.01** |
| **MC(ZJ)** | **2.0 (2.0-2.0)** | **1.0 (1.0-1.0)** | **0.01** |
| **MC(XZ)** | **2.0 (2.0-2.0)** | **1.0 (1.0-1.0)** | **0.01** |
| **MC(JX)** | **2.0 (2.0-2.0)** | **1.0 (1.0-1.2)** | **0.02** |
| **MC(HE)** | **2.0 (2.0-2.0)** | **1.1 (1.0-1.7)** | **0.04** |
| **MC(CQ)** | **1.8 (1.0-2.0)** | **1.0 (1.0-1.0)** | **0.01** |
| **MC(FJ)** | **1.7 (1.0-2.0)** | **1.0 (1.0-1.0)** | **0.01** |
| MC(NM) | 1.4 (1.0-2.0) | 1.0 (1.0-1.0) | 1.00 |
| MC(HA) | 1.2 (1.0-2.0) | 1.0 (1.0-1.0) | 1.00 |
| MC(GS) | 1.0 (1.0-1.0) | 1.0 (1.0-1.0) | 1.00 |
| MC(HI) | 1.0 (1.0-1.0) | 1.0 (1.0-1.0) | 1.00 |
| MC(HLJ) | 1.0 (1.0-1.0) | 1.0 (1.0-1.0) | 1.00 |
| MC(JL) | 1.0 (1.0-1.0) | 1.0 (1.0-1.0) | 1.00 |
| MC(SC) | 1.0 (1.0-1.0) | 1.0 (1.0-1.0) | 1.00 |
| MC(LN) | 1.0 (1.0-1.0) | 1.0 (1.0-1.0) | 1.00 |
| MC(TJ) | 1.0 (1.0-1.0) | 1.0 (1.0-1.0) | 1.00 |
| MC(NX) | 1.0 (1.0-1.0) | 1.0 (1.0-1.0) | 1.00 |
| MC(QH) | 1.0 (1.0-1.0) | 1.0 (1.0-1.0) | 1.00 |
| MC(SX) | 1.0 (1.0-1.0) | 1.0 (1.0-1.0) | 1.00 |

**
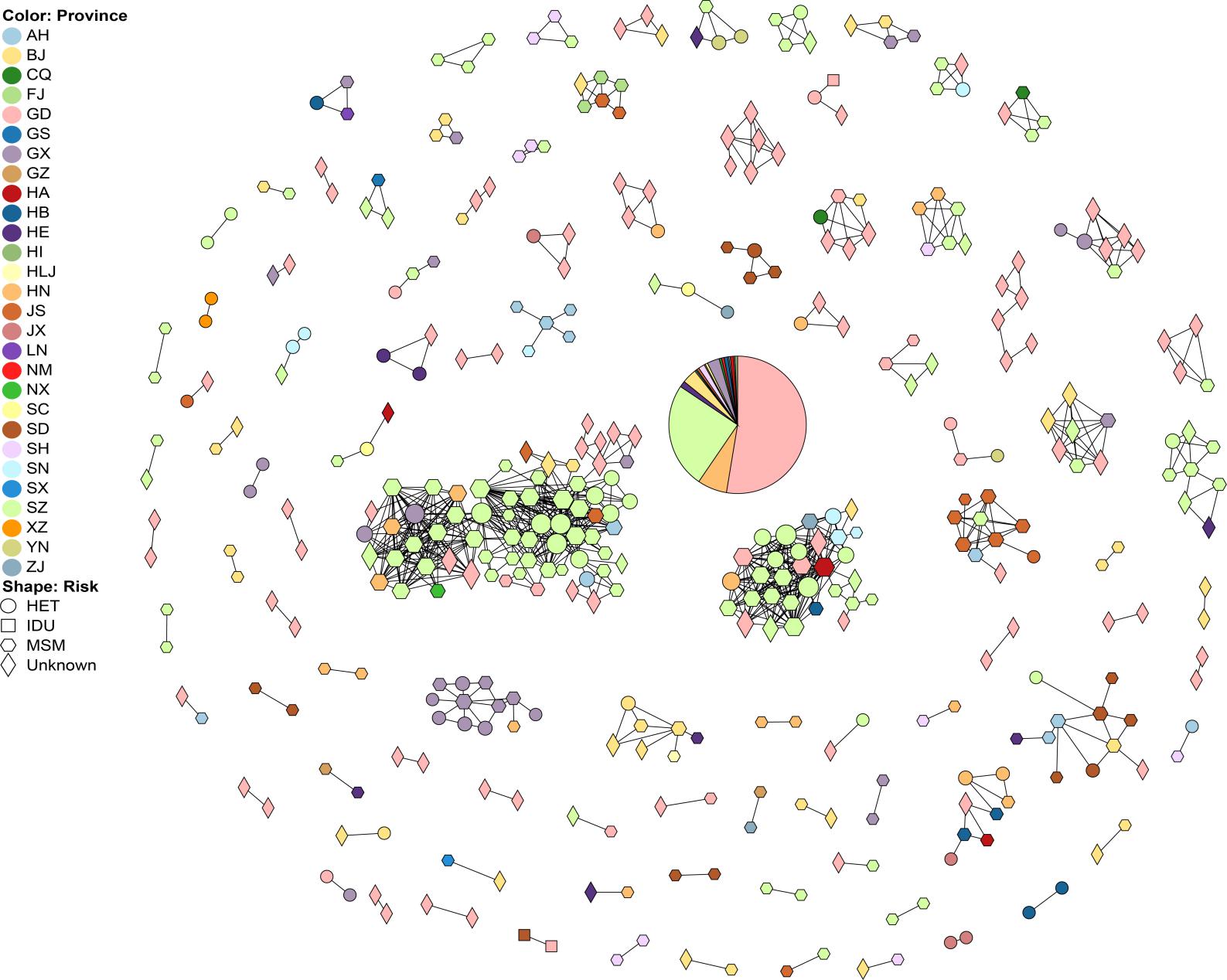
**

**Figure S1.** The molecular network diagram of CRF55_01B strain. The pie chart in the figure represents the largest molecular cluster in the molecular network. Clusters of 56.1% (55/98) were found in only a single region, 36.7% (36/98) clusters of South, 9.2% (9/98) clusters of North, 6.1% (6/98) clusters of East, 3.1% (3/98) clusters of Center and 1.0% (1/98) cluster of Southwest. Clusters of 44.9% (44/98) were found in only a single province, 17.3% (17/98) clusters of GD, 8.2% (8/98) clusters of SZ, 8.2% (8/98) clusters of BJ, 3.1% (3/98) clusters of SD, 2.0% (2/98) clusters of GX, 2.0% (2/98) clusters of HN, 1.0% (1/98) cluster of SH, 1.0% (1/98) cluster of JX, 1.0% (1/98) cluster of HB and 1.0% (1/98) cluster of XZ. 44.9% (44/98) of clusters were MSM, 15.3% (15/98) of clusters were HET, and 18.4% (18/98) of clusters included MSM and HET. It was worth noting that clusters with nodes≥10 all contained MSM and HET.

**
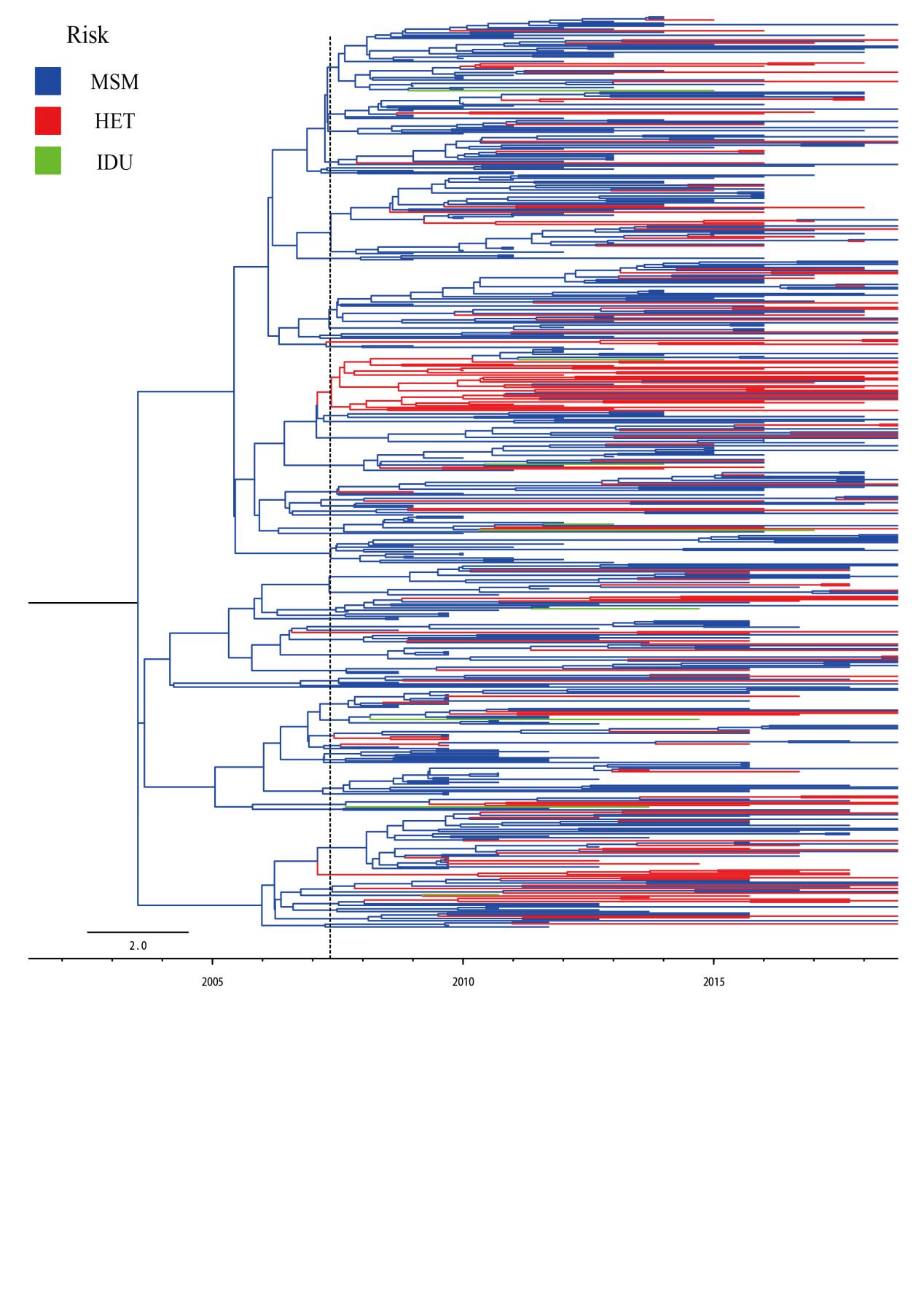
**

**Figure S2.** The MCC trees of CRF55_01B strain in the risk. The dotted line in the figure shows that CRF55_01B originated from MSM and began to spread to HET around 2007.4 (95% HPD interval: 2006.5-2008.1).
